# Phylogeography and population structure of *Lagocephalus spadiceus* (Richardson, 1845) (Tetraodontiformes, Tetraodontidae) in the South China Sea

**DOI:** 10.1002/ece3.11320

**Published:** 2024-04-25

**Authors:** Hao Xu, Liangliang Huang, Tao Chen, Caiguang Wang, Zhiqiang Wu, Yanan Cheng, Qiongyuan Su, Bin Kang, Yunrong Yan, Xiuguo Zhang

**Affiliations:** ^1^ College of Environmental Science and Engineering Guilin University of Technology Guilin China; ^2^ Guangxi Key Laboratory of Theory and Technology for Environmental Pollution Control Guilin China; ^3^ Collaborative Innovation Center for Water Pollution Control and Water Safety in Karst Areas Guilin China; ^4^ College of Basic Medicine Guilin Medical University Guilin China; ^5^ Fisheries College Ocean University of China Qingdao China; ^6^ Fisheries College Guangdong Ocean University Zhanjiang China; ^7^ Guangxi Jinggong Marine Science and Technology Ltd Beihai China

**Keywords:** *COI* and Cyt *b* gene datasets, genetic diversity, *Lagocephalus spadiceus*, phylogeography, South China Sea

## Abstract

The climate fluctuations during the Late Pleistocene significantly influenced the phylogeographic structure and historical dynamics of marine fishes in the marginal seas of the western Pacific Ocean. The puffer fish, *Lagocephalus spadiceus*, holds substantial nutritional and economic value in the South China Sea. To investigate the demographic history and population structure of the *L. spadiceus*, the mitochondrial DNA *COI* and Cyt *b* gene datasets from 300 individuals across eight populations in the South China Sea were sequenced. Our findings revealed high haplotype diversity (0.874 ± 0.013) and low nucleotide diversity (0.00075 ± 0.00058). The phylogenetic tree and haplotype networks revealed no significant genetic differentiation along the northern coast of South China Sea. Neutrality tests, mismatch distribution analyses, and Bayesian skyline plots suggested that *L. spadiceus* underwent population expansion during the Late Pleistocene. Both ocean currents and climate change significantly influenced the geographical distribution and genetic population structure of *L. spadiceus*.

## Graphical abstract

We examined the phylogeographic pattern and demographic history of *L. spadiceus* for the first time, using 300 individuals from eight populations along the coast of mainland China and Hainan Island. In addition, the new date of the sequence could provide useful information for phylogeography and evolutionary studies.

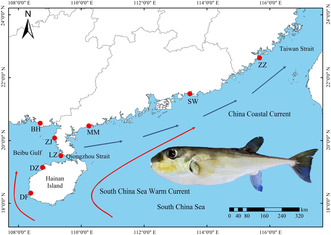

## 1 INTRODUCTION

In marine species, the phylogeography and genetic differentiation were influenced by historical events, including ocean current systems, vicariance, Pleistocene climatic cycles, and life‐history characteristics of organisms (Ding et al., 2018; Liu et al., 2007). It is worth noting that some marine fishes have fragile genetic structures due to their extensive larval and adult dispersal (Ashrafzadeh et al., 2021; Caccavo et al., 2018). Climate oscillations during the Pleistocene greatly altered the environment of marginal seas of the western Pacific, including the South China Sea (SCS). During glacial periods, the SCS formed a semi‐enclosed sac‐shaped gulf and exposed approximately 0.7 million km^2^ of continental shelf (Wang & Sun, 1994). Previous molecular studies have shown that many marine fishes with high mobility exhibit low genetic structure in the SCS, such as *Cirrhimuraena chinensis* (Li et al., 2014) and *Nuchequula mannusella* (Gao et al., 2019). The phylogeographic study of marine fishes alive in the SCS has particular significance for interpreting the consequences of past events, geological configurations, and modern oceanographic aspects in this environment (He et al., 2010).

*Lagocephalus spadiceus* is a non‐toxic *Lagocephalus* species (Tuney, 2016), belonging to Tetraodontiformes, Tetraodontidae, and *Lagocephalus*. It is a nearshore warm‐water demersal fish that inhabits depths between 3 and 200 m (Tuncer et al., 2008), distributed along the southern coast of Africa in the Indian Ocean, eastward to Indonesia and the Philippines of the Pacific Ocean, and northward to the coast of China (Liu et al., 2016). In China, it occurs along the coastal areas of the SCS. Almost *L. spadiceus* is imported from China in Japan (Yamaguchi et al., 2013), but this also makes it an easy target for widespread exploitation. Recently, since the continuous increase in fishing intensity and deteriorating environmental conditions, the wild resources of *L. spadiceus* have been drastically reduced (Hardy et al., 2014). There are fewer reports on germplasm resources and genetic diversity evaluation of *L. spadiceus*. To better protect and rationally develop the wild germplasm resources of *L. spadiceus*, it is imperative to conduct a genetic diversity assessment to establish a theoretical basis for the scientific conservation and sustainable utilization of its genetic resources.

Genetic diversity is a vital component of biodiversity and a prerequisite for the continuous adaptation of species or populations to environmental change and survival evolution. Species with a higher genetic diversity possess a greater ability to environmental changes (Roldan et al., 2000). Mitochondrial DNA (mtDNA) constitutes a tiny fraction of organismal genome size but has been widely used as a marker of molecular diversity in animals for the past four decades (Galtier et al., 2009). This tool has been widely embraced by population geneticists, following the works of Avise et al. (1987) and Moritz et al. (1987), among others. Experimentally, mtDNA is present in most cells with high copy numbers and is relatively easy, rapid, and inexpensive to sequence (Zink & Barrowclough, 2008). Due to the relationship between the rate and time of evolution, effective information sites are different, and their resolving power is different. Therefore, concatenating *COI* and Cyt *b* markers can increase the number of effective genetic sites, resulting in more accurate and effective information compared to single gene analysis (Barrientos‐Villalobos & Schmitter‐Soto, 2019; Halasan et al., 2021).

Our study examines the phylogeography, population genetic diversity, and demographic history of *L. spadiceus* using *COI* and Cyt *b* gene datasets. As of current knowledge, there has not been a documented report on the population genetic of *L. spadiceus*. This study aims to address the lack of information on *L. spadiceus* in the region by providing a comprehensive background report. Meanwhile, our study has contributed to a better understanding of the evolutionary process that has influenced the phylogeography of coastal marine fishes in China.

## 2 MATERIALS AND METHODS

### 2.1 Sample collection

**FIGURE 1:**
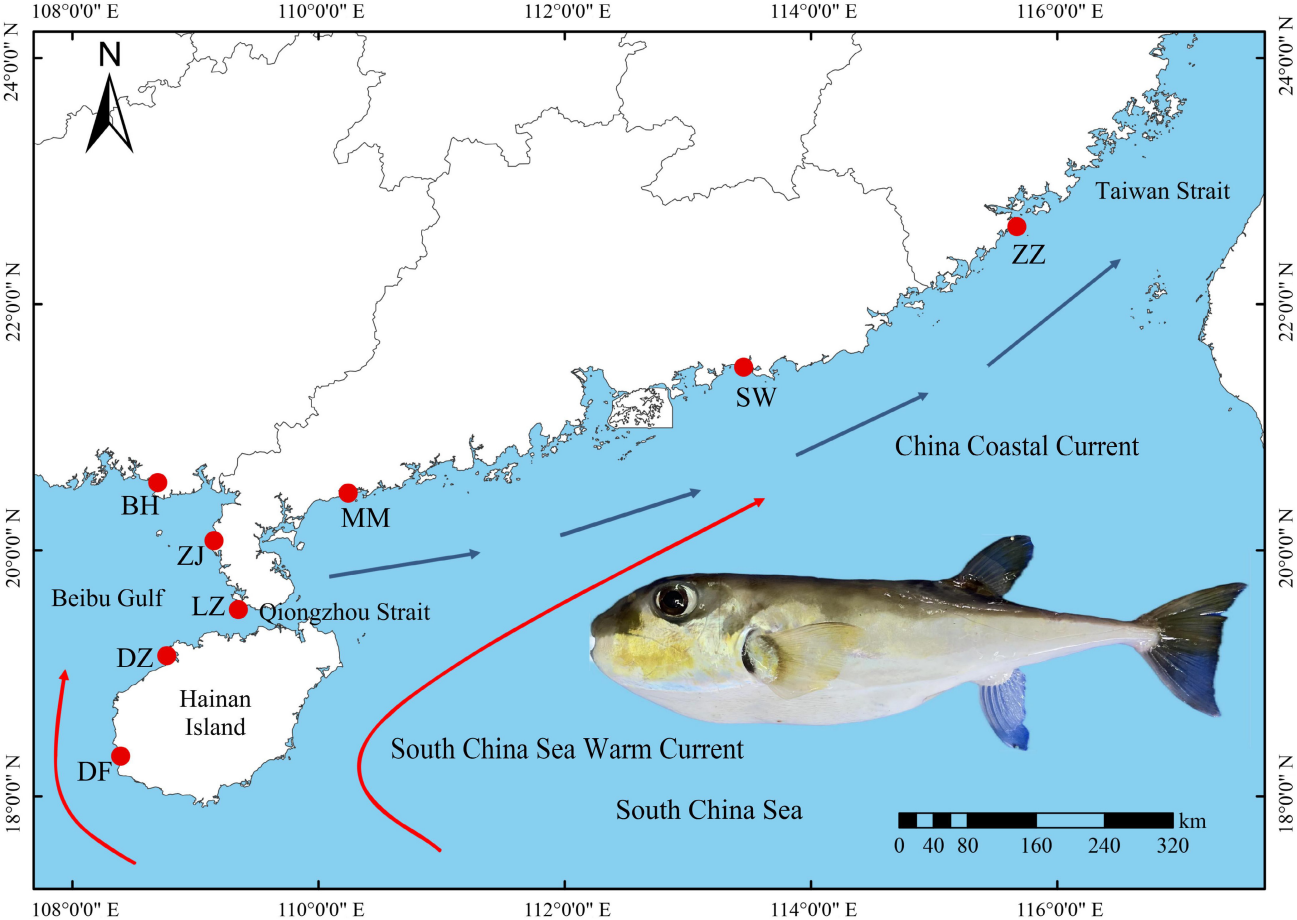
Map of sampling geographic locations for *Lagocephalus spadiceus*. The map was downloaded from the National Geomatics Center of China with modifications using Arcgis10.7. In spring and summer, the China Coastal Current (blue arrow) and South China Sea Warm Current (red arrow) flow northward into the East China Sea (Wang et al., 2015). *Note*: BH, Beihai; ZJ, Zhanjiang; LZ, Leizhou; DZ, Danzhou; DF, Dongfang; MM, Maoming; SW, Shanwei; ZZ, Zhangzhou.

A total of 300 specimens of *L. spadiceus* were obtained from eight geographic locations, including Beihai (BH), Zhanjiang (ZJ), Leizhou (LZ), Danzhou (DZ), Dongfang (DF), Maoming (MM), Shanwei (SW), and Zhangzhou (ZZ) in the South China Sea (Figure 1, Table 1). All *L. spadiceus* specimens were collected through bottom trawl surveys from April 2022 to April 2023. These were specifically for the purpose of investigating the structure of fishery resources in the Beibu Gulf and its adjacent seawaters. A morphological analysis was used to identify species (head, dorsal surface, and ventral surface are covered with small spines, the dorsal side of the body is brownish‐yellow or yellow‐green, and caudal fin is white at the upper and lower tips) (Chen & Zhang, 2015; Liu et al., 2016). Fishes were given access to muscle tissue, which was then stored in 1.5 mL vials with 95% ethanol at −20°C until genomic extraction. The experimental animal care and use adhered strictly to the guidelines, policies, and laws concerning animal welfare as stipulated by the Animal Research and Ethics Committees of the Guilin University of Technology (Approval Code: GLUT‐EAEC2021010) animal welfare laws, guidelines, and policies.

**TABLE 1:** Sampling locations and descriptive statistics of genetic diversity of *L. spadiceus.*

Population code	*D*	*N*	*S*	*H*	*H*_d_	*π*	*K*
BH	April 2022	43	27	18	0.859 ± 0.046	0.00081 ± 0.00025	2.131
ZJ	August 2022	53	19	19	0.867 ± 0.030	0.00065 ± 0.00016	1.702
LZ	August 2022	33	22	14	0.817 ± 0.052	0.00070 ± 0.00021	1.827
DZ	August 2022	33	11	11	0.761 ± 0.082	0.00061 ± 0.00011	1.595
DF	August 2022	32	17	15	0.867 ± 0.053	0.00071 ± 0.00017	1.852
MM	September 2022	35	15	17	0.911 ± 0.030	0.00078 ± 0.00014	2.057
SW	April 2023	30	16	14	0.842 ± 0.060	0.00069 ± 0.00016	1.803
ZZ	September 2022	41	20	15	0.854 ± 0.037	0.00072 ± 0.00018	1.892
Total		300	94	95	0.874 ± 0.013	0.00075 ± 0.00058	1.953

*Note*: *D*, sampling date; *N*, sample size; *S*, number of polymorphic sites; *H*, number of haplotypes; *H*_d_, haplotype diversity (±SD); *π*, nucleotide diversity (±SD); and *K*, mean pairwise difference.

### 2.2 DNA extraction, PCR amplification, and sequencing

The total DNA of *L. spadiceus* was extracted from each muscle following the FastPure Cell/Tissue DNA Isolation Mini Kit (Nanjing, China). The purity and concentration of DNA were checked using an ultra‐microspectrophotometer (NanoDrop 2000, United States of America). The primers of mtDNA were adapted from Li et al. (2018). The *COI* was boosted by the primers *COI‐*F: 5′‐AAACCACCGCCTGACACTC‐3′ and *COI‐*R: 5′‐GGGATTTTAACCCCCGGCAT‐3′, the Cyt *b* was boosted by the primers Cyt *b*‐F: 5′‐ GCGCCCCAAAGTAAGGAGAA‐3′ and Cyt *b*‐R: 5′‐ GGGATTTTAACCCCCGGCAT ‐3′. PCR amplification volume of 50 μL, including 25 μL 2 × *Taq*PCR Master Mix, 2 μL each of primers (10 μmol/L), 1 μL DNA template, and 20 μL ddH_2_O. PCR cycling conditions were applied: initial denaturation at 94°C for 5 min, 35 cycles of denaturation at 94°C for 1 min (*COI*) or 30 s (Cyt *b*), annealing at 58°C (*COI*) or 56°C (Cyt *b*) for 1 min, extension at 72°C for 1 min, and final elongation at 72°C for 8 min (*COI*) or 5 min (Cyt *b*). Every PCR product was electrophoresed on 1% agarose gel, and PCR products were sent to Guangzhou Ige Biotechnology Ltd (Guangzhou, China) for purification and DNA sequencing.

### 2.3 Data analysis

#### 2.3.1 Genetic diversity

Forward and reverse splicing of all sequences using SeqMan in Larsergene v7.1.0 (Swindell & Plasterer, 1997), and then compare and edit the sequences using the Clustal W method in MEGA v7.0 (Kumar et al., 2016). The *COI* and Cyt *b* sequences were matched one by one for multi‐locus sequence analysis (MLSA) using PhyloSuite v1.2.2 (Zhang et al., 2020). Count base composition and content, polymorphic sites (*S*), and parsimony informative sites (*P*_*i*_) using MEGA v7.0 (Kumar et al., 2016). The haplotype numbers (*H*), haplotype diversity (*H*_d_), nucleotide diversity (*π*), and mean pairwise difference (*K*) were counted using DnaSP v6.0 (Rozas et al., 2017).

#### 2.3.2 Genetic structure

The Bayesian information criterion (BIC) in jModelTest v2.1.10 (Darriba et al., 2012) was used to establish a substitution model for the haplotype datasets before phylogenetic analysis. Subsequently, the mitochondrial *COI* and Cyt *b* gene dataset haplotypes were used to reconstruct the phylogenetic tree using the Bayesian inference (BI). The congeneric species *Lagocephalus laevigatus* was chosen as an outgroup, from the NCBI access number NC_015345.1 (*COI* and Cyt *b*). Bayesian inference study was carried out using MrBayes v3.2.7 (Ronquist et al., 2012), and one set of four chains was permitted to run concurrently for 20 million generations. Every 1000 generations, a sample of the tree was taken, with the first 25% being eliminated as burn‐in. As the sampled generations increased, the log likelihood maintained a constant level, and stationarity was attained when the split average frequencies' average standard deviation was less than 0.01 (Hall, 2016). Phylogenetic tree editing was done with FigTree v1.4.4 (http://tree.bio.ed.ac.uk/software/figtree/), and a median‐joining haplotype network was produced using PopART v1.7 (Leigh & Bryant, 2015).

Genetic distance within and between populations was calculated using MEGA v7.0 (Kumar et al., 2016). Subsequently, AMOVA was used to quantify genetic variation using *F*‐statistics at two geographically distinct levels of subdivision: among and within populations. To test for statistical significance, 10, 000 permutations of the fixation index *F*_ST_ were performed between pairs of populations using Arlequin v3.5 (Excoffier & Lischer, 2010).

#### 2.3.3 Demographic history

The Tajima's *D* (Tajima, 1989) and Fu's *F*s (Fu & Li, 1993) tests were utilized to check for neutral evolution. To examine population growth, the mismatch distribution (Rogers & Harpending, 1992) between the sum of squared deviations (SSD) and Harpending's raggedness index (Rg) was analyzed with Arlequin v3.5 (Excoffier & Lischer, 2010). Changes in effective population size (*Ne*) over time were deduced using Bayesian skyline plot analysis in BEAST v2.6.3 (Bouckaert et al., 2019). To account for possible site‐specific variations, the rate of clock mutation was fixed at 1 × 10^−8^ per year, as recommended for reef fishes (Delrieu‐Trottin et al., 2017). With a sample every 1000 iterations, 100 million generations of separate independent Markov chain Monte Carlo (MCMC) studies were carried out. The molecular clock was calibrated using an average divergence rate of 2% per million years for mtDNA (Schubart et al., 1998). ESS values were detected until they reached 200, and these parameter values were displayed in Tracer v1.7.1 (Rambaut et al., 2018).

## 3 RESULTS

### 3.1 Genetic diversity

**FIGURE 2:**
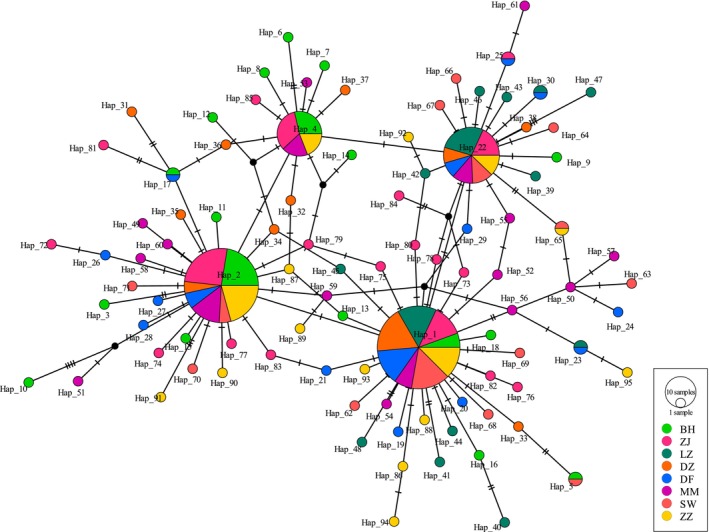
Median‐joining network for *COI* and Cyt *b* gene datasets haplotypes of *Lagocephalus spadiceus*. The size of the circles is proportional to haplotype frequency.

A total of 300 *COI* and Cyt *b* gene datasets (2621 bp) were obtained of *L. spadiceus* from eight geographic locations (Figure 1). A total of 94 polymorphic sites were detected among all individuals, including 28 parsimony information sites and 66 singleton variable sites (Table 1). The average nucleotide composition was 23.2% adenine (A), 27.3% thymine (T), 31.6% cytosine (C), and 17.9% guanine (G), with a slightly higher content of A+T (50.5%) than G+C (49.5%), showing a clear anti‐G bias. The mutation of DNA was unsaturated for the transition/transversion (Ts/Tv) of bases in the *COI* and Cyt *b* gene datasets, which was 3.73. A total of 95 haplotypes were identified (GenBank accession numbers: OQ970201‐OQ970253 and OR428269‐OR428309), the majority of which were unique haplotypes (89.47%). Only 10 haplotypes were shared between populations (Hap_1, Hap_2, Hap_4, Hap_5, Hap_17, Hap_22, Hap_23, Hap_25, Hap_30, and Hap_65), and the most common Hap_1 (24.0%), Hap_2 (19.3%), and Hap_22 (11.0%) were found at each population (Figure 2).

**FIGURE 3:**
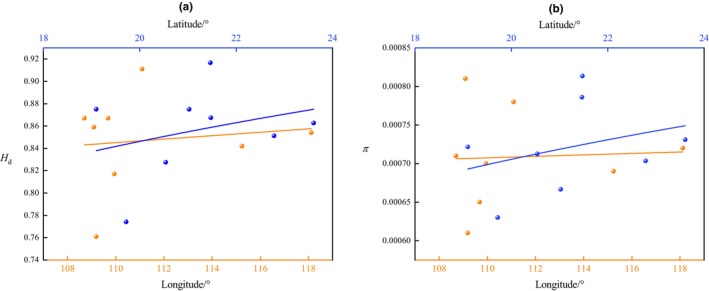
Plots and trendlines of haplotype diversity (*H*_d_) and nucleotide diversity (*π*) with longitude (yellow line) and latitude (blue line).

Total haplotype diversity (*H*_d_) was high (*H*_d_ = 0.874 ± 0.013), while nucleotide diversity (*π*) was low (*π* = 0.00075 ± 0.00058) (Table 1), showing the high haplotype diversity and low nucleotide diversity. The correlation between genetic diversity and longitude and latitude of sampling locations showed that the haplotype diversity (*r* = .29) and nucleotide diversity (*r* = .31) of *L. spadiceus* populations tend to rise with latitude but did not change significantly with longitude (*r* = .08 and .04, respectively) (Figure 3).

### 3.2 Genetic structure and differentiation

The molecular evolution model with the gamma shape parameter (HKY+I+G) was found to be the best substitution model for the *COI* and Cyt *b* gene datasets by the jModelTest. From this result, a BI tree was built to determine phylogenetic relationships across populations (Figure S1). The BI tree was dispersed with haplotypes from each population and lacked well‐supported groups. Neither significant genealogy branches nor haplotype clusters could be identified about the sampling locations.

Network analysis and the phylogenetic tree showed similar results. The connection between several haplotypes resembled a star, with certain prominent haplotypes like Hap_1, Hap_2, Hap_4, and Hap_22 (Figure 2). Unnoticeable clades in the network diagram of reticulations do not correlate with sampling locations, suggesting a substantial gene flow among populations and recent population expansion. It was found that relationships between populations were not linked to geological networks, but instead to the haplotypes that were present in each population. According to these results, there was no obvious phylogeographical pattern of *L. spadiceus* in the South China Sea.

The degree of genetic variation between populations was evaluated using *F*_st_ pairwise comparisons. The *F*_st_ values were typically low and even negative, as shown in Table 2; only the *F*_st_ values between the LZ population and other populations were higher and significant (*p* < .05). The genetic distance between and within populations was at the same level, with little differentiation (Table 2). The range under investigation had no significant genetic structure. Hierarchical AMOVA analysis revealed that genetic variation in all populations existed within populations, while only a small proportion of genetic variation could be attributed to differences between populations (Table 3).

**TABLE 2:** Genetic distance and differentiation between pair populations of *L. spadiceus.*

Population	BH	ZJ	LZ	DZ	DF	MM	SW	ZZ
BH	0.00081	0.00074	0.00098	0.00077	0.00085	0.00082	0.00066	0.00079
ZJ	0.0122	0.00065	0.00080	0.00064	0.00070	0.00071	0.00072	0.00074
LZ	0.2259*	0.1554*	0.00070	0.00070	0.00075	0.00085	0.00071	0.00082
DZ	0.0735*	0.0167	0.0707*	0.00061	0.00065	0.00071	0.00065	0.00067
DF	0.1007*	0.0374*	0.0590*	−0.0143	0.00071	0.00076	0.00069	0.00072
MM	0.0190	−0.0055	0.1281*	0.0121	0.0194	0.00079	0.00078	0.00075
SW	0.1407*	0.0720*	0.0276	0.0017	−0.0066	0.0508*	0.00069	0.00068
ZZ	0.0251*	−0.0052	0.1279*	0.0014	0.0100	−0.0087	0.0437*	0.00072

*Note*: Genetic distance among population (above the diagonal), genetic distance within population (diagonal), and genetic differentiation coefficient (below the diagonal), significant level *p < .05.

**TABLE 3:** Summary of hierarchical analysis of molecular variances for *L. spadiceus.*

Grouping	Variance components	% variation	*F*‐statistics	*p*‐Value
For all groups				
Group 1 (BH, ZJ, LZ, DZ, DF, MM, SW, ZZ)	Among groups in total	94.58	*F*_ST_: 0.0542	.0000
Divided by the Qiongzhou Strait				
Group 1 (BH, ZJ, LZ, DZ, DF)	Among groups	−1.90	*F*c_T_: −0.0190	.0174
	Among populations within groups	6.48	*F*_SC_: 0.0636	.0000
Group 2 (MM, SW, ZZ)	Within populations	95.42	*F*_ST_: 0.0458	.0000
Divided by the mainland and Hainan Island				
Group 1 (BH, ZJ, LZ, MM, SW, ZZ)	Among groups	−1.12	*F*c_T_: −0.0113	.0381
Group 2 (DZ, DF)	Among populations within groups	5.87	*F*_SC_: 0.0580	.0000
	Within populations	95.26	*F*_ST_: 0.0474	.0000

### 3.3 Demographic history

**FIGURE 4:**
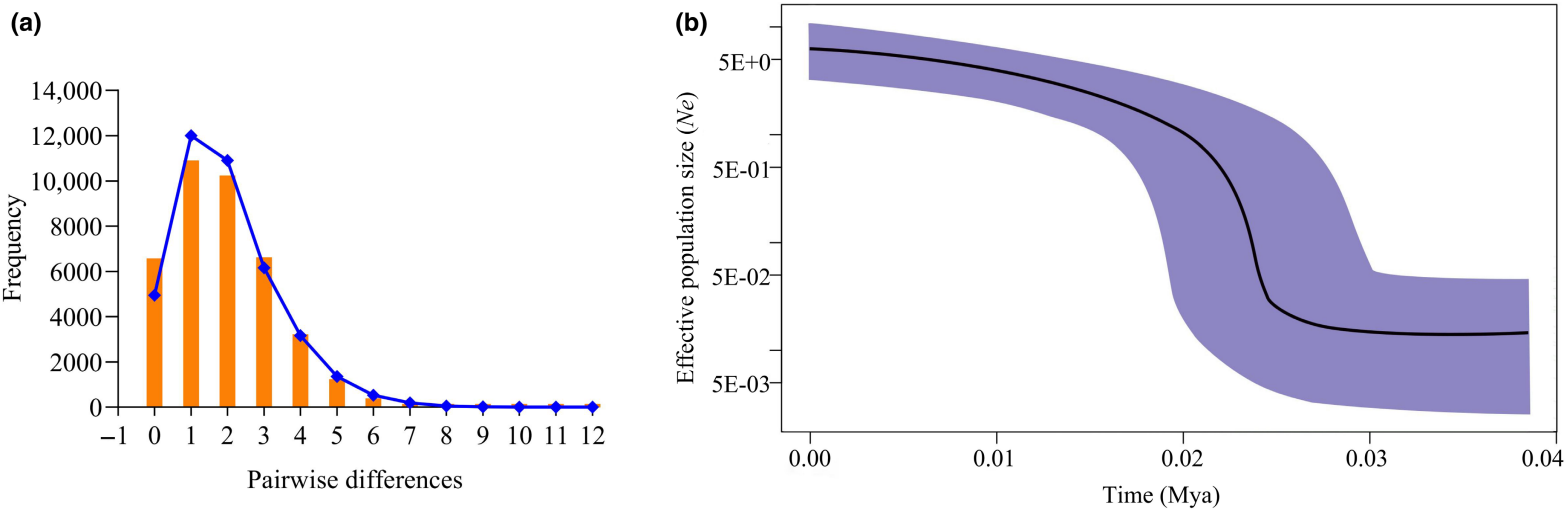
Mismatch distribution (a) and Bayesian skyline plots (b) for *COI* and Cyt *b* gene datasets of *Lagocephalus spadiceus*. In the Bayesian skyline plots, the *X*‐axis represents time using a mutation rate of 2% per million years ago (Mya) (Schubart et al., 1998), and the *Y*‐axis represents the effective population size (*Ne*). The two approaches reveal obvious signals of demographic expansion for the South China Sea. The mean estimate and both 95% highest posterior density (HPD) limits are indicated.

A unimodal distribution was observed in the mismatch distribution analysis (Figure 4a), which was consistent with the expected distribution under a sudden expansion model (Rg = 0.0536, *p* > .05, Table 4). It is also possible that populations had undergone expansion in the past (as indicated by the star‐like networks). The sum of the squared deviation (SSD) showed that there was no significant deviation from the growth and expansion model (*p* > .05). The Tajima's *D* and Fu's *F*s tests of *L. spadiceus* were significantly negative (Tajima's *D* = −1.905, *p* < .05; Fu's *F*s = −10.543, *p* < .01, Table 4). Typically, such values indicated that *L. spadiceus* may have experienced population expansion.

**TABLE 4:** Neutrality tests and mismatch distribution parameter estimates for *L. spadiceus.*

Population	Tajima's *D*	Fu's *F*s	SSD	Rg	tau
BH	−2.299**	−12.911**	0.0008	0.0337	1.507
ZJ	−1.874*	−14.857**	0.0032	0.0558**	1.228
LZ	−2.314**	−8.782**	0.0023	0.0254	0.937
DZ	−1.453	−5.981**	0.0048	0.0526	1.506
DF	−1.971**	−11.101**	0.0091	0.0618	1.421
MM	−1.443	−12.446**	0.0010*	0.0467*	2.448
SW	−1.906*	−9.526**	0.0116	0.0908	1.776
ZZ	−1.976**	−8.737**	0.0061	0.0617*	1.428
Total	−1.905*	−10.543**	0.0049	0.0536	1.531

Abbreviations: Rg, Harpending's Raggedness Index; SSD, sum of squared deviation; tau, expansion parameter.

Significant level *p < .05, **p < .01.

Bayesian skyline plots supported demographic scenarios explaining the recent population expansion of *L. spadiceus* (Figure 4b). The population experienced a significant increase, followed by a period of demographic stability. The calculated population expansion time is approximately from 0.025 to 0.010 Mya during the Late Pleistocene (Figure 4b).

## 4 DISCUSSION

### 4.1 Genetic diversity

Haplotype diversity (*H*_d_) and nucleotide diversity (*π*) are two important indicators to measure genetic diversity, and *π* represents the proportion of each haplotype in the populations, which can reveal the polymorphism of mtDNA in the populations more accurately (Chen et al., 2022). Grant and Bowen (1998) concluded that *H*_d_ was higher than 0.5 and *π* was greater than 0.005, which indicated higher species diversity. This study revealed high levels of haplotype and low levels of nucleotide diversity, which is common among some marine fish species (Avise et al., 1987; Zhang et al., 2006).

High genetic diversity plays a crucial role in the exploitation and restoration of fishery resources (DeWoody et al., 2021). Assessing genetic diversity is an effective approach to the adaptability and survival ability of species in response to environmental changes (Schmitt & Hewitt, 2004), which is essential for species management and conservation. The fish of *L. spadiceus* is an economic species and has been caught for a long time. Compared with other marine fishes in the same sea area, *L. spadiceus* had a lower nucleotide diversity (Niu et al., 2019; Xu et al., 2021; Yi et al., 2021), reflecting that *L. spadiceus* population in the South China Sea has a fragile genetic diversity and requires conservation as well as a sustainable development planning from fishery management.

### 4.2 Genetic structure and differentiation

Genetic distance is a crucial factor in determining the genetic relationship between species (Mather et al., 2017). Shaklee et al. (1982) proposed a classification of fish genetic distance at the population level (0.05), species level (0.30), and genus level (0.90). In this study, it was found that the genetic distance between populations was small, indicating a close genetic relationship between these populations. According to the coalescent theory (Crandall & Templeton, 1993), more diverse populations have longer coalescence times and larger coalescent effective population sizes than less diverse populations (assuming the same mutation rate). Therefore, the ancestral haplotype was the most widely distributed. In the *COI* and Cyt *b* gene datasets, Hap_1 and Hap_2 were found to be the dominant haplotypes (Figure 2) and may be the origin of *L. spadiceus*.

In the phylogenetic analysis, the haplotypes of eight populations were randomly distributed. The haplotype network and phylogenetic tree also showed no clear pedigree structure corresponding to geographical location. This pattern suggests that the species went through a bottleneck event followed by a population expansion (Grant & Bowen, 1998). The dispersal of larvae with ocean currents is an important cause of the limited genetic differentiation of marine fishes that have a geographically large distribution range (Strathmann et al., 2002). In this study, *L. spadiceus* were caught in the spring and autumn, during this time, the China Coastal Current and the South China Sea Warm Current flowed northward into the East China Sea (Figure 1) (Wang et al., 2015; Yang et al., 2008). Previous studies have reported that extensive gene exchange occurs over a wide geographical range in marine fishes (Grant & Bowen, 1998; Niu et al., 2019; Yi et al., 2021). *F*_st_ is a significant measure in evaluating genetic diversity among populations (Holsinger & Weir, 2009). A higher *F*_st_ value suggests a greater level of genetic differentiation. According to Wright (1951) classification, *F*_st_ value of 0–0.05 suggests no differentiation, 0.05–0.15 suggests little differentiation, 0.15–0.25 suggests moderate differentiation, and *F*_st_ value greater than 0.25 suggests significant genetic differentiation. The *F*_st_ between the BH and LZ populations was the highest value among all populations (Table 2), indicating that the greatest genetic differentiation was between these populations, and other populations were lower.

### 4.3 Demographic history

This study utilized the Tajima's *D*, Fu's *F*_S_ tests, and mismatch analysis to suggest that a population expansion event of the *L. spadiceus* population may have occurred from 0.025 to 0.010 Mya in the Late Pleistocene. *L. spadiceus* is mainly distributed less than 50 m depth, and spawns in coastal habitats and shallow shorelines. Therefore, the *L. spadiceus* distribution is closely related to historical sea level fluctuations. During the Last Glacial Maximum of the Pleistocene, the northern South China Sea encompassed the Beibu Gulf, a segment of the South China continent. This region was connected to Hainan Island, while Taiwan Island was linked to mainland China. The entire South China Sea was separated from the Indian Ocean to form a semi‐closed basin (Wang, 1990). The survival range of marine fish decreased sharply; therefore, the *L. spadiceus* population may have moved and survived in one or more glacial refuges during this period, such as the semi‐closed South China Sea. In the Late Pleistocene, the glaciation began to disappear and *L. spadiceus* might have experienced rapid population expansion when favorable conditions occurred.

Many studies have demonstrated a weak genetic differentiation between the geographical populations of surface marine fish that can migrate long distances or swim. This can be attributed to the free dispersal of floating eggs, fish larvae, juveniles, and adults, as well as the absence of significant geographical obstacles in the open ocean environment. Consequently, gene exchange occurs extensively and widely among these marine fish populations (Canfield et al., 2022; Hewitt, 2000; Palumbi, 1994). However, it should be noted that *L. spadiceus*, being a benthic fish, does not exhibit a long‐distance migration behavior according to its life history. Therefore, the observed panmixia among populations may be attributed to their early life habits. The active diffusion of fish larvae and juveniles as well as marine environmental factors, such as ocean circulation and climate change in the Late Pleistocene, have played crucial roles in shaping the systematic geographical pattern and population genetic structure of *L. spadiceus*.

### 4.4 The choice of mtDNA

Surprisingly, despite the popularity of mtDNA as a marker in evolutionary studies, this assumption only relies on a handful of comparisons involving mostly vertebrate species (Nabholz et al., 2009). Depending on the species, the mtDNA mutation rate was much higher or lower than the nuclear DNA (nuDNA) rate. For example, it is not always clonal, far from neutrally evolving and certainly not clock‐like, and the ratio of mitochondrial to nuclear mutation rate varies widely among animals (Allio et al., 2017; Galtier et al., 2009). Despite these long‐acknowledged concerns, similar results were obtained in several studies that have employed mtDNA and nuDNA to investigate genetic structuring and demographic history in populations of marine fishes (Adams et al., 2006; Machado‐Schiaffino et al., 2009; Mccusker & Bentzen, 2010; Vinas et al., 2010; Yang et al., 2022).

## 5 CONCLUSIONS

In this study, we present the first exploration of the genetic structure of *L. spadiceus* in the SCS. The mtDNA sequences analysis of specimens from the SCS revealed no significant genetic differentiation among sampling sites, with low *F*_st_ values indicating genetic homogeneity, which probably reflected widespread and recent historical interconnections during the post‐glaciation. Hainan Island and Leizhou Peninsula did not affect the gene flow of *L. spadiceus* in the SCS. In its demographic history, it experienced a low effective population size during the Quaternary period that increased sharply after the Last Glacial Maximum. The phylogeographic pattern of *L. spadiceus* may be attributed to past population expansion and long‐distance larval dispersal facilitated by present‐day ocean currents. Mitochondrial DNA (mtDNA) markers, which are maternally inherited, solely represent a singular evolutionary history. Consequently, they fail to provide an accurate representaion of the overall population structure. To enhance our understanding of the population structure of *L. spadiceus*, it is imperative to conduct further research utilizing more precise nuclear genetic markers such as microsatellites, single nucleotide polymorphisms, and other mtDNA genes. These markers enhance the efficacy of molecular data in substatiating phylogeographic hypotheses, offering supplenentary insights into population structure.This information can subsequently guide the formulation of n effective management policy and contribute to the conservation of fish resources.

## AUTHOR CONTRIBUTIONS

**Hao Xu:** Conceptualization (equal); data curation (equal); formal analysis (equal); investigation (equal); methodology (equal); resources (equal); software (equal); validation (equal); writing – original draft (equal); writing – review and editing (equal). **Liangliang Huang:** Conceptualization (equal); formal analysis (equal); funding acquisition (equal); project administration (equal); resources (equal); supervision (equal); writing – review and editing (equal). **Tao Chen:** Formal analysis (equal); methodology (equal); supervision (equal); visualization (equal); writing – review and editing (equal). **Caiguang Wang:** Data curation (equal); investigation (equal). **Zhiqiang Wu:** Data curation (equal); funding acquisition (equal); supervision (equal). **Yanan Cheng:** Formal analysis (equal); software (equal). **Qiongyuan Su:** Data curation (equal); investigation (equal). **Bin Kang:** Formal analysis (equal); funding acquisition (equal); supervision (equal); writing – review and editing (equal). **Yunrong Yan:** Funding acquisition (equal); writing – review and editing (equal). **Xiuguo Zhang:** Funding acquisition (equal); project administration (equal).

## FUNDING INFORMATION

This work is funded by the Key Research and Development Program of Guangxi (Guike AB22035050), National Natural Science Foundation of China (U20A2087), and Guangxi Key Laboratory of Beibu Gulf Marine Biodiversity Conservation, Beibu Gulf University (2022KA01).

## CONFLICT OF INTEREST STATEMENT

The authors declare that they have no competing interests.

## Supporting information


Figure S1

## Data Availability

The haplotype DNA sequences were deposited in GenBank under accession numbers OQ970201‐OQ970253 (*COI*) and OR428269‐OR428309 (Cyt *b*). The data have been uploaded into Dryad under the following https://doi.org/10.5061/dryadd.cjsxksnc3.
