# Supplementary figures and images for: Phylogeography and population structure of Lagocephalus spadiceus (Richardson, 1845) (Tetraodontiformes, Tetraodontidae) in the South China Sea
Source: Ecol Evol. 2024 Apr 25;14(4):e11320. doi: 10.1002/ece3.11320 (PMC11045559; doi:10.1002/ece3.11320)

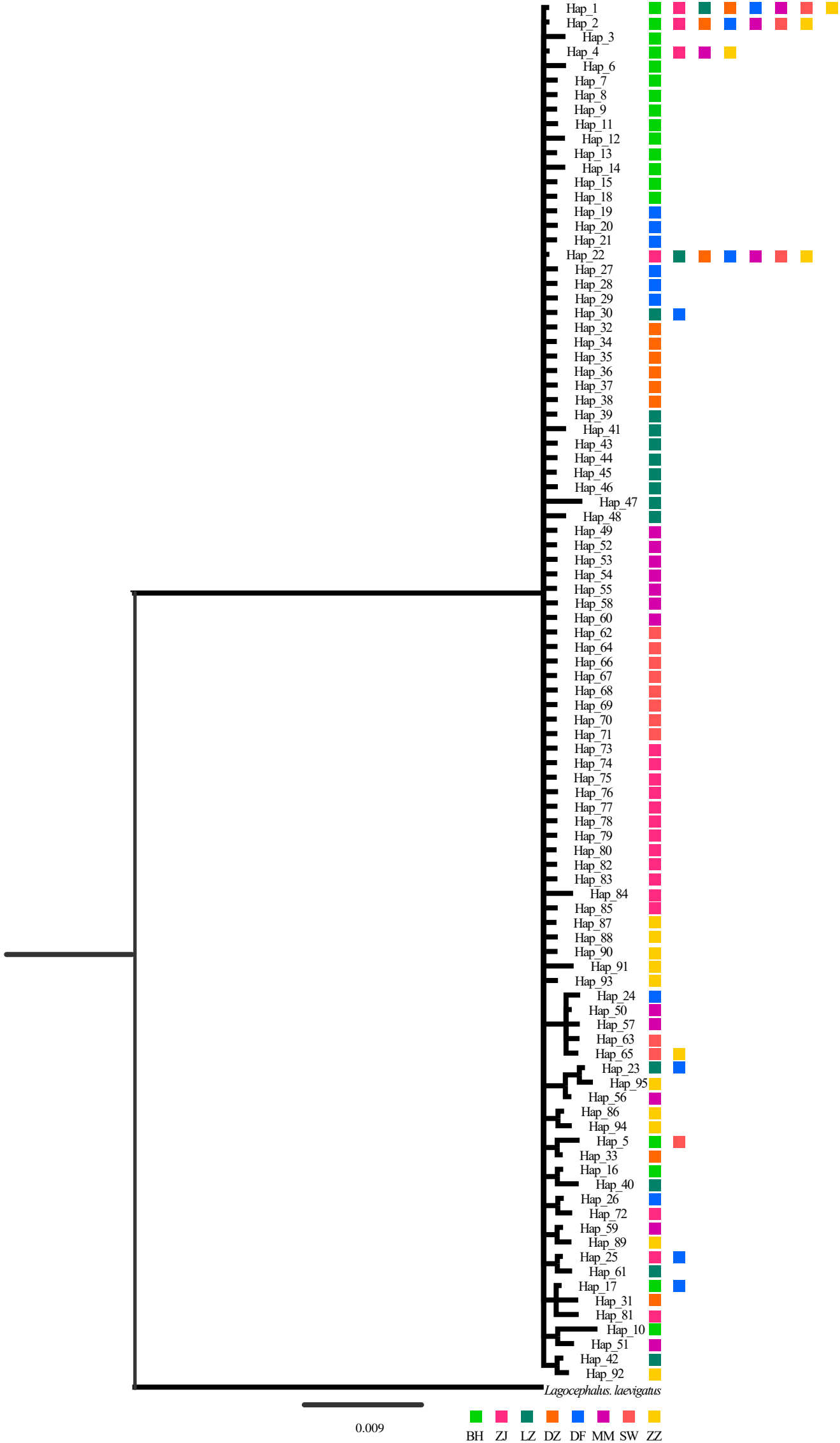

Supplement: Supplementary file 1 — Figure S1 [file ECE3-14-e11320-s001.pdf]
